# Incidence of Rib Fracture following Treatment with Proton Therapy for Breast Cancer

**DOI:** 10.14338/IJPT-22-00034.1

**Published:** 2023-03-24

**Authors:** Julie A. Bradley, Xiaoying Liang, Raymond B. Mailhot Vega, Chunbo Liu, Eric D. Brooks, Teena Burchianti, Emma Viviers, Roi Dagan, Oluwadamilola T. Oladeru, Christopher G. Morris, Nancy P. Mendenhall

**Affiliations:** 1 Department of Radiation Oncology, University of Florida College of Medicine, Gainesville and Jacksonville, FL, USA; 2 Mayo Clinic, Jacksonville, FL, USA; 3 Department of Radiation Oncology, The First Affiliated Hospital of Zhengzhou University, Zhengzhou, China; 4 University of Florida Health Proton Therapy Institute, Jacksonville, FL, USA

**Keywords:** breast cancer, adverse events, particle therapy

## Abstract

**Purpose:**

To determine the rib fracture rate in a cohort of patients with breast cancer treated with proton therapy.

**Patient and Methods:**

From a prospective database, we identified 225 patients treated with proton therapy between 2012 and 2020 (223 women; 2 men). Clinical and dosimetric data were extracted, the cumulative incidence method assessed rib fracture rate, and Fine-Gray tests assessed prognostic significance of select variables. In-field rib fracture was defined as a fracture that occurred in a rib located within the 10% isodose line. Out-of-field rib fracture was defined as a fracture occurring in a rib location outside of the 10% isodose line.

**Results:**

Of the patients, 74% had left-sided breast cancer; 5%, bilateral; and 21%, right-sided. Dual-energy x-ray absorptiometry scans showed normality in 20%, osteopenia in 34%, and osteoporosis in 6% (test not performed in 40%). Additionally, 57% received an aromatase inhibitor. Target volumes were breast ± internal mammary nodes (IMNs) (16%), breast and comprehensive regional lymphatics (32%), chest wall ± IMNs (1%), and chest wall/comprehensive regional lymphatics (51%). Passive-scattered proton therapy was used for 41% of patients, 58% underwent pencil-beam scanning (PBS), and 1% underwent a combination (passive scattering/PBS), with 85% of patients receiving a boost. Median follow-up was 3.1 years, with 97% having >12-month follow-up. The 3-year cumulative in-field rib fracture incidence was 3.7%. Eight patients developed in-field rib fractures (1 symptomatic, 7 imaging identified) for a 0.4% symptomatic rib fracture rate. Median time from radiation completion to rib fracture identification was 1.8 years (fractures were identified within 2.2 years for 7 of 8 patients). No variables were associated with rib fracture on univariate analysis. Three fractures developed outside the radiation field (0.9% cumulative incidence of out-of-field rib fracture).

**Conclusion:**

In this series of patients with breast cancer treated with proton therapy, the 3-year rib fracture rates remain low (in-field 3.7%; symptomatic 0.4%). As in photon therapy, the asymptomatic rate may be underestimated owing to a lack of routine surveillance imaging. However, patients experiencing symptomatic rib fractures after proton therapy for breast cancer are rare.

## Introduction

Optimizing the therapeutic ratio is a consistent goal across oncology care. In radiation therapy, treatment plans are optimized for individual patients through the selection of beam arrangements, beam energy, radiation technique, and type of radiation beam. Proton therapy has dosimetric advantages, primarily in cardiac sparing but also in improving target coverage [1–4]. Proton beams have a higher linear energy transfer (LET) at the end of their range [5–7]; in breast cancer treatment, the beams end in the lungs, ribs, muscle, or soft tissue.

Radiation therapy carries the risk of bone injury, including fractures and necrosis; in immature bones, it can cause hypoplasia. In breast cancer management with radiation therapy, the ribs are in close proximity to the target volume and, in some cases, even included in the target volume. The published rates of rib fracture after radiation therapy vary by radiation modality and treatment era. Herein we report the rate of rib fracture in a cohort of patients enrolled in a prospective registry and treated with proton therapy for breast cancer.

## Materials and Methods

We identified patients treated with proton therapy for breast cancer at our institution between January 1, 2012, and December 31, 2020. All included patients signed a consent form to participate in an institutional review board (IRB)–approved prospective registry (IRB 201702651). Patients who received > 1 fraction per day, those undergoing re-irradiation or partial breast irradiation, and those treated with matched photon-proton fields were excluded from this analysis. Clinical and dosimetric data were extracted from the electronic medical record with the primary intent to assess the rate of rib fracture in patients with breast cancer treated with proton therapy. Rib fracture was assessed prospectively as part of routine follow-ups in the radiation oncology department as part of a VisionTree Optimal Care (VTOC) assessment performed for every patient at every follow-up. A retrospective chart review of the medical records was also performed to ensure no fractures were missed on the VTOC assessment and to capture rib fractures that may have occurred since the patient's prior follow-up in radiation oncology. SAS version 9.4 (Cary, North Carolina) and JMP Pro version 16.0 (Cary, North Carolina) were used for statistical analysis. The cumulative incidence method assessed the rib fracture rate; the Fine-Gray test statistic assessed the prognostic significance of select variables.

A 4-dimensional (4D) computed tomography (CT) scan was obtained for CT simulation, with contouring and treatment planning performed on the average reconstruction for all 10 phases of the 4D CT scan. Target contours for regional nodal irradiation were drawn per the RTOG (Radiation Therapy Oncology Group) or RADCOMP (Radiotherapy Comparative Effectiveness) atlases [8,9]; the chest wall contour excluded the ribs and intercostal muscles, and the internal mammary and supraclavicular contours were continuous without a gap. The posterior edge of the clinical target volume (CTV) chest wall aligned with the anterior edge of the ribs, whereas the anterior edge of the pectoralis muscle was the posterior boundary for the CTV breast. For the chest wall target, 3 mm was subtracted from the body surface to create the CTV chest wall, while a 5-mm subtraction was used for intact breasts. The ribs were not prospectively contoured as an organ at risk, and a rib dose constraint was not used in the planning process. Ribs were contoured retrospectively for the patients who experienced rib fracture(s). For those patients, the ribs on the treated side were contoured on CT imaging performed during posttreatment surveillance and on which the rib fractures were visible. That CT image set was fused with the treatment planning scan, and the dose was overlaid to allow measurement of the dose to the rib at the site of fracture(s).

Passive-scattered proton therapy was used for all patients from 2012 through mid-2017. The technique has been described in a previous publication [10]. In mid-2017, pencil-beam scanning (PBS) became available at our institution and became the preferred treatment approach for patients with breast cancer. The treatment plans were created in the RayStation treatment planning system (RaySearch Laboratories, Stockholm, Sweden). Two anterior oblique beam angles between 0° and 40° were used. A water-equivalent 7.4-cm Lexan ranger shifter was used for each beam. The plan was robustly optimized with a setup uncertainty of 5 mm and a 3.5% range uncertainty. The Monte Carlo algorithm was used for optimization with a sampling history of 50 000 ions/spot, and the final dose was computed with 0.5% statistical uncertainty.

Per our institutional follow-up guidelines, patients were evaluated 1 month after radiation, then every 6 months for 5 years, and then annually through year 10. Patients underwent surveillance mammography. Imaging with CT, positron emission tomography, and/or x-ray was performed as needed, based on patient symptoms or individual risk assessment and disease characteristics. In-field rib fracture was defined as a fracture that occurred in a rib located within the 10% isodose line. Out-of-field rib fracture was defined as a fracture occurring in a rib location outside of the 10% isodose line.

## Results

A total of 225 patients were identified in our prospective cohort, including 223 women and 2 men. The median age at the time of radiation was 57.8 years (range, 25-86.8). Demographically, 26% were Black patients, 69% were White patients, 5% were of Hispanic ethnicity, and 5% were other races (or the patient's race was not disclosed). Of these patients, 74% had left-sided breast cancer, 5% had bilateral breast cancer, and 21% had right-sided breast cancer. A total of 134 of the 225 patients underwent bone density evaluation with dual-energy x-ray absorptiometry (DEXA). Of those who underwent testing, the results were normal in 32.8% (n = 44), while 56.7% (n = 76) had osteopenia and 10.4% (n = 14) had osteoporosis. In addition, 23.6% of this cohort received bisphosphonates, and 57.3% received antiendocrine therapy with an aromatase inhibitor. Vitamin supplementation with calcium was used by 41.3% of patients, and vitamin D by 66.7% of patients; 31.1% of patients had a diagnosis of vitamin D insufficiency or deficiency (≤ 30 ng/mL). The median body mass index (BMI) for this cohort was 25.8 kg/m^2^ (range, 16.9-53.5). In addition, 36% of patients had a BMI ≥ 30%, meeting the definition of obesity. The breast ± IMNs were treated in 16.4% of patients, the breast and comprehensive regional lymphatics in 31.6%, the chest wall ± IMNs in 1.3%, and the chest wall and comprehensive regional lymphatics in 50.7%.

In all, 41% of patients were treated with passive-scattered proton therapy (n = 92); 52% of these underwent a mastectomy. In all, 58% of patients received treatment with PBS proton therapy (n = 131); 53% of these underwent a mastectomy. A combination of passive scattering and PBS was used for 2 patients (1%) owing to machine availability.

In all, 85.3% of patients received a sequential boost to the lumpectomy cavity, mastectomy incision, or chest wall (for inflammatory disease), and/or nodal region (such as gross IMNs that were not resected). The boost was delivered with passive-scattered proton therapy in 26.6% of patients, PBS in 49.5%, electrons in 16.1%, and photons in 5.2%. Three patients (1.6%) underwent boost treatments using both electron (to the mastectomy incision) and passive-scattered proton therapy (to the nodal regions). In addition, 1 patient received intraoperative radiation therapy before whole breast proton therapy. Hypofractionation was used in 11.1% of patients (dose, 40.05-42.4 GyRBE [Gray, relative biological effectiveness] in 15-16 fractions), all of whom had breast-conserving surgery, while 80.9% of patients were treated with 2 GyRBE per fraction for a median of 25 fractions (range, 23-25). In the earlier years, 8% of patients were treated with 50.4 GyRBE at 1.8 GyRBE per fraction in 28 daily fractions. Boost doses ranged from 4 to 20 Gy or GyRBE in 2 to 10 fractions, with a median of 10 Gy.

**Figure 1.**
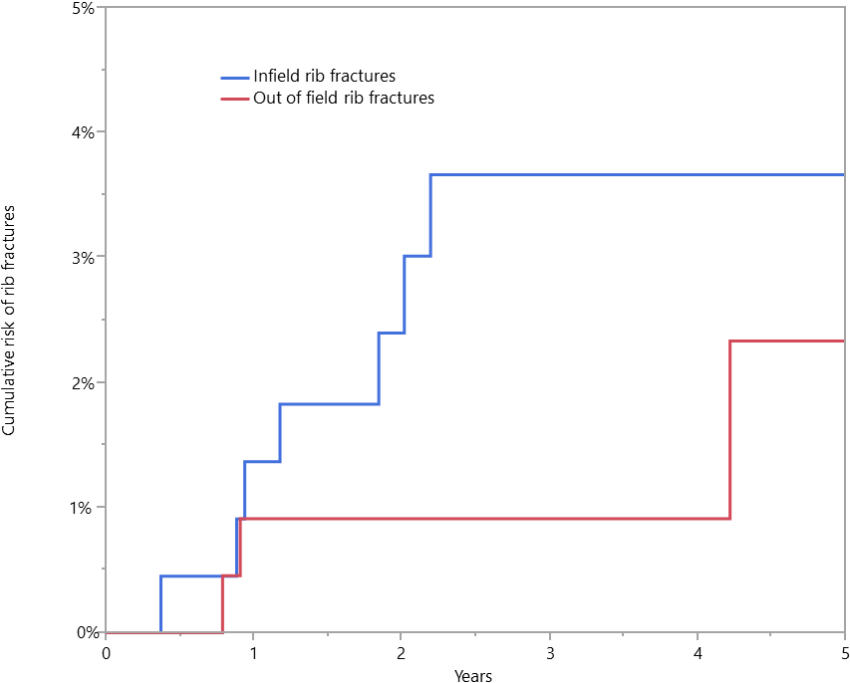
Cumulative incidence function of rib fractures for both in-field and out-of-field fractures.

The median follow-up was 3.1 years (range, 0.2-9.1), with 96.5% of patients having > 12 months of follow-up. The 3-year cumulative incidence of patients with in-field rib fracture was 3.7% (95% confidence interval [CI], 1.6%-7.1%) (**Figure 1**). In total, 8 patients developed in-field rib fractures, 1 symptomatic, and 7 identified incidentally on surveillance imaging, for a 0.4% rate of symptomatic rib fracture. Of those affected, patients had a range of 1 to 6 ribs fractured, with a median of 2 ribs fractured. Three of the 8 patients had trauma, with imaging evaluation demonstrating fractures. Two of these 3 patients had fractures in other areas. **Table 1** provides the patient and treatment characteristics of those who developed a rib fracture in the radiation field. The symptomatic patient, a 40-year-old White woman with normal bone density and a BMI of 30.5 kg/m^2^ who was treated with passive-scattered proton therapy, initially had chest wall pain with negative imaging, then experienced a fall with fractures elsewhere. Imaging later showed 2 in-field rib fractures.

**Table 1.** Patient and treatment characteristics of patients who developed a rib fracture in the radiation field (n = 8).

**Characteristic**	**Patient**							
	**1**	**2**	**3**	**4**	**5**	**6^a^**	**7**	**8**
Age, y	59	51	30	61	48	40	75	68
BMI, kg/m^2^	22.7	25.2	18.2	30.9	22.7	30.5	24.7	28.0
Race	Black	White	Subject declined	White	White	White	White	White
Breast cancer laterality	Right	Right	Left	Right	Right	Right	Left	Bilateral
DEXA results	Not tested	Normal	Abnormal; “below the expected range for her age”	Osteopenia	Not tested	Normal	Normal	Osteoporosis
Use of a bisphosphonate	No	No	No	No	No	Yes	No	Yes
Use of an aromatase inhibitor	No	Yes	No	Yes	Yes	No	Yes	Yes
Diagnosis of vitamin D insufficiency/deficiency	No	No	No	No	No	No	Yes	No
Calcium supplementation	No	Yes	No	No	Yes	No	Yes	Yes
Vitamin D supplementation	Yes	Yes	No	Yes	Yes	Yes	Yes	Yes
Target volume	Breast + RNI	Breast + RNI	Breast + RNI	CW (no reconstruction) + RNI	CW (immediate expander reconstruction) + RNI	CW (immediate expander reconstruction) + RNI	Breast + RNI	Breast + RNI
Type of radiation therapy: initial phase	Photon (5 fractions)/PBS (20 fractions)	Passive- scattered	PBS	Passive- scattered	PBS	Passive- scattered	PBS	PBS
Total dose: initial phase, Gy	50	50.4	50	50	50	50	50	50
Type of radiation therapy: boost phase	Photon	Passive- scattered	PBS	Passive- scattered	Electrons	Passive- scattered	PBS	PBS
Total dose: boost phase, Gy	10	16	10	10	10	10	20	0
Time from end of RT to rib fracture, y	2.1	7.4	1.7	2.2	0.4	0.9	0.9	1.8
Number of fractures	3	1	4	6	2	2	2	1
Appearance on imaging	Old	Subacute to chronic	Nonacute	New (since CT chest done at 18 mo after RT)		Linear lucencies with the appearance of fracture	Subacute	Age - indeterminate
Rib fracture in boost area?	Yes	Unknown	Yes	Yes	∼ 50% of boost dose to area of fracture	No	Yes	N/A
D0.5cm^3^ to any rib in the radiation field, Gy	66.1	69.0	59.9	61.9	50.7	53.0	68.7	50.8
D0.5cm^3^ to any of the fractured ribs, Gy	60.5	50.3	58.8	59.9	47.4	52.8	59.6	38.3
Additional notes	Two falls in the year rib fractures were diagnosed, resulting in a broken ankle and broken wrist.	-	-	Inflammatory breast cancer; grade 3 chest wall fibrosis after RT; grade 1 pulmonary fibrosis (radiographic)	-	Pain in the inferior right chest wall 6 mo after RT. Chest x-ray imaging and chest CT were negative for rib fracture and other etiology. She then experienced a fracture of the fibula and medial malleolus after a fall. PET-CT 3 mo later demonstrated the in-field rib fractures	-	Diagnosed on imaging following motor vehicle accident

**Abbreviations:** BMI, body mass index; DEXA, dual-energy x-ray absorptiometry; RNI, regional node irradiation; CW, chest wall; PBS, proton pencil-beam scanning; RT, radiation therapy; CT, computed tomography; N/A, not applicable; D0.5cm^3^, the minimal dose received by the highest irradiated volumes of 0.5 cm^3^; PET, positron emission tomography.

a Denotes patient with symptomatic rib fracture.

**Figure 2.**
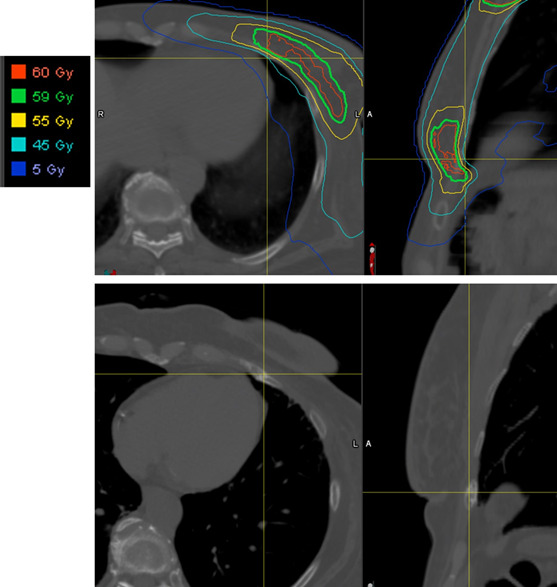
The dose distribution of the PBS plan is displayed in the top panels, with the rib that later fractured shown in the bottom panel. This rib was in the field of the lumpectomy boost, with a D0.5cm^3^ dose of 59.6 Gy. Abbreviations: D0.5cm^3^, the minimal dose received by the highest irradiated volumes of 0.5 cm^3^; PBS, proton pencil-beam scanning.

**Figure 3.**
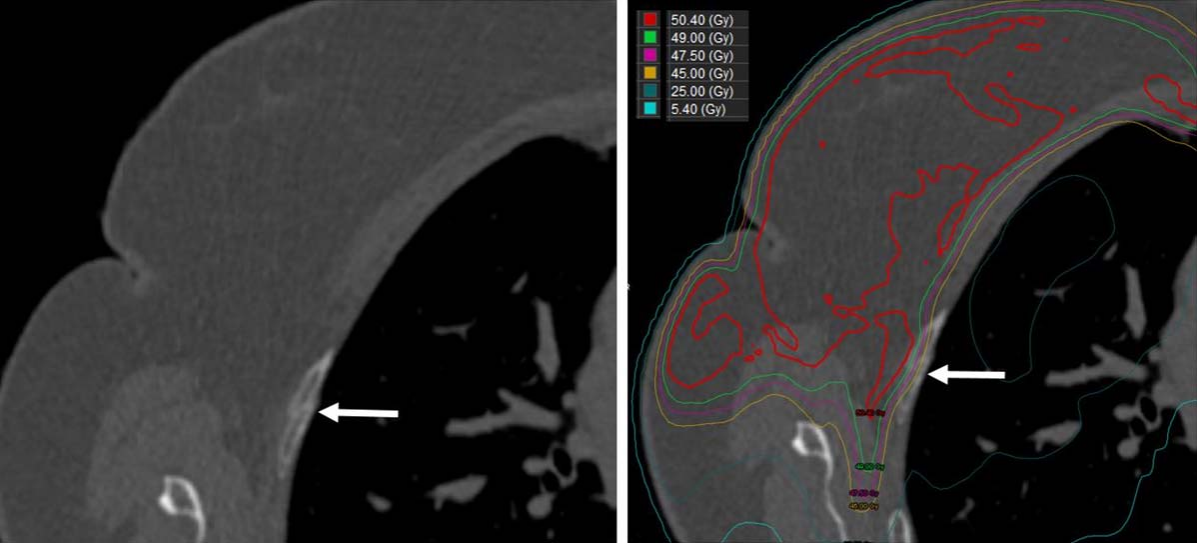
The right third rib was fractured (arrow) after the patient experienced a motor vehicle accident (left). The radiation dose was deformed to the CT that demonstrated this fracture (right). The D0.5cm^3^ dose to the fractured rib was 38.3 Gy. Abbreviations: CT, computed tomography; D0.5cm^3^, the minimal dose received by the highest irradiated volumes of 0.5 cm^3^.

The median time from completion of radiation to the identification of rib fracture was 1.8 years (range, 0.4-7.4), with only 1 fracture discovered after 2.2 years. The median D0.5cm^3^ (the dose to 0.5 cm^3^ volume of rib) to any fractured rib was 55.8 GyRBE (range, 38.3-60.5). Interestingly, the ribs that fractured were not necessarily the ribs with the highest dose exposure. For all ribs in the radiation field, the median D0.5cm^3^ dose was 60.9 GyRBE (range, 50.8-69). **Figures 2** and **3** demonstrate the dosimetry and incidence of subsequent rib fractures for 2 patients who experienced an in-field fracture.

To estimate the biological dose delivered to the ribs after considering the increased RBE at the proton beam distal edge, we analyzed 4 patient cases. All of these 4 patients received comprehensive nodal irradiation with an initial dose of 50 Gy and a boost dose of 10 Gy at 2 Gy per fraction. In addition, these 4 patients' boost volumes were all in the proximity of the rib. Therefore, these 4 patients were selected, as they represented the cases with the highest dose to the ribs. The dose-averaged linear energy transfer (LETd)–weighted dose was calculated by using the linear RBE model published by McMahon et al [11]. A detailed description of the LETd dose calculation can be found in our previous publications [12, 13]. The D0.5cm^3^ rib dose ranged from 59.5 to 60.9 Gy when assuming a constant RBE of 1.1. However, the LETd-weighted dose increased owing to the increased RBE at the end of the range and ranged from 64.1 to 68.5 Gy.

On univariate analysis, no patient or treatment-related factors were statistically significant, including patient age at the time of radiation, race, ethnicity, use of an aromatase inhibitor, supplementation with calcium and/or vitamin D, diagnosis of vitamin D deficiency, or type of proton radiation therapy (passive-scattered versus PBS) (**Table 2**). Three patients with in-field rib fractures had a low bone density, 3 had a normal bone density, and 2 were untested. All patients had a BMI < 31 kg/m^2^, with 75% of affected patients being White.

**Table 2.** Risk of in-field rib fracture by patient variable.

**Variable**	**5-Year fracture rate, %**	**95% L**	**95% U**	***P*** **value**
Age, y				.53
<50	4.2	1.1	10.7	
≥50	3.3	1.1	7.8	
Race				.42
Black	2.1	0.2	9.9	
Other	3.9	1.4	8.4	
Type of proton therapy				.36
DS	2.4	0.4	7.5	
PBS	4.7	1.7	10.2	
Use of aromatase inhibitor				.72
No	3.7	0.9	9.5	
Yes	3.7	1.2	8.6	
Calcium supplementation				.70
No	3.8	1.2	8.9	
Yes	3.5	0.9	9.1	
Vitamin D supplementation				.22
No	1.5	0.1	7.0	
Yes	4.7	1.9	9.5	
Vitamin D insufficiency				.30
No	4.6	1.9	9.3	
Yes	1.4	0.1	7.0	
Type of surgery				.52
Lumpectomy	4.2	1.4	9.7	
Mastectomy	3.1	0.8	8.2	
Treatment of IMN				.18
No	0.0	0.0	0.0	
Yes	4.3	1.9	8.2	
Boost (any type)				.25
No	0.0	0.0	0.0	
Yes	4.4	1.9	8.4	
DEXA result				.46
Abnormal	4.1	1.1	10.6	
Normal	4.4	0.8	13.5	
Receipt of chemotherapy				.28
No	1.6	0.1	7.6	
Yes	4.4	1.8	8.9	

**Abbreviations:** 95% L, lower confidence interval; 95% U, upper confidence interval; DS, double-scatter proton therapy; PBS, pencil-beam scanning proton therapy; IMN, internal mammary node; DEXA, dual-energy x-ray absorptiometry.

Three patients developed rib fractures outside of the radiation field, for a cumulative incidence of out-of-field rib fracture of 0.9% (95% CI, 0.2%-3.0%) (**Figure 1**). These fractures occurred 0.8, 0.9, and 4.2 years after radiation therapy. Two with out-of-field rib fractures had osteopenia, and 1 did not have DEXA testing.

## Discussion

Rib fractures have been known as a potential toxicity resulting from breast cancer radiation therapy since 1955 [14]. In addition to radiation, other cancer therapies for breast cancer increase the risk of rib fractures, including endocrine therapy and chemotherapy-induced menopause [15, 16]. Patient factors can also contribute; age, weight, race, and smoking and alcohol use are known variables affecting bone density [17].

In this series of 225 patients treated with proton therapy for breast cancer, the 3-year cumulative incidence of patients with symptomatic rib fracture was only 0.4%, while the overall asymptomatic and symptomatic in-field rib fracture incidence was 3.7%. During the same period, 3 patients developed rib fractures outside of the radiation field, for a 3-year actuarial rate of out-of-field rib fracture of 0.9%, suggesting an absolute increase in asymptomatic and symptomatic rib fracture above the baseline of 2.8%. This rate is consistent with the photon series. Robinson et al [18] surveyed women treated for breast cancer > 5 years before self-reported “minimal trauma fractures,” finding a fracture rate of 13.6% overall, with ribs as the most common (though not the only) site of fracture. In that study, receipt of radiation therapy was not associated with rib fracture, while osteoporosis and postmenopausal status were shown to correlate with rib fractures. In 1988, Overgaard [19] reported on 231 patients treated with postmastectomy radiation who underwent surveillance chest x-ray imaging 1 to 6 years after radiation. Asymptomatic rib fracture incidence varied with fraction size, reaching 19% with ∼ 4 Gy per fraction compared to 6% with ∼ 2 Gy per fraction. In 2002, Meric et al [20] reported a retrospective review of long-term outcomes of irradiation after breast-conserving surgery, with a median follow-up of 7.4 years and a rib fracture rate of 0.3% (1 of 294). Patients were treated between 1990 and 1992 with either cobalt or on a linear accelerator, in which photons were primarily used but a medial electron field to target the IMN chain was common. Fung et al [21] published a 1% (1 of 110) rate of rib fracture in patients receiving 6-MV photons to a median dose of 64 Gy for bilateral breast cancer, with a median follow-up of > 4 years. For these 2 studies, details were not provided regarding specifics of follow-up or imaging, or distinguishing asymptomatic versus symptomatic rib fractures. In a retrospective study of bone scans for patients who had undergone mastectomy and adjuvant radiation therapy (4-6 MV photons to a dose of 50.4 Gy), the rib fracture rate identified on imaging was 1.4% (4 of 292) with a 4.5-year median follow-up [22]. In an older series of radiation therapy alone for breast cancer, the 3-year rate of rib fracture was 1% for doses < 70 Gy, but this increased to 6% for doses > 85 Gy [23]. This rate of 6% for doses > 85 Gy supports the dose estimation by Wang et al [7] for LETd with proton therapy. While measurement of LETd is not currently available at our institution, we observed a wide range of doses to 0.5 cm^3^ of the fractured rib, including 1 patient with a dose < 40 Gy. In addition, the location of the rib fracture was often not in the location of the highest rib exposure, suggesting a multifactorial etiology, perhaps including rib anatomy, the type of inciting event contributing to the fracture, and individual patient risk factors. The extent of radiation contribution to in-field rib fractures remains to be elucidated.

Data for the incidence of rib fracture after proton therapy are emerging. Jimenez et al [24] reported a 7% rate of rib fracture (5 of 69) after treatment with PBS, with a median time to fracture of 15.9 months. All rib fractures were grade 1 per CTCAE (Common Terminology Criteria for Adverse Events), version 4.0 (US National Cancer Institute; Bethesda, Maryland). Patients did not undergo routine imaging surveillance; incidental rib fractures identified on imaging for other indications were included. In combining their prospective PBS series with a retrospective proton series, the rib fracture rate was 6.4% (13 of 203) [7]. Our series included patients treated with both passive-scattered proton radiation and PBS; the rib fracture rate was notably lower at 3.7%, with a slightly longer median time to rib fracture (21 months). This series included proton therapy with both passive scattering and PBS, with no difference in the rates of rib fracture between these modalities on univariate analysis. Verma et al [25] reported on 91 patients with breast cancer treated with uniform scanning (77%) or PBS (23%). With a median follow-up of 15.5 months, the crude rate of rib fractures was 2%, with 1 patient developing a rib fracture 13 months after radiation and the other 39 months after radiation. The rib fractures were not categorized as symptomatic or asymptomatic. Imaging after treatment was performed as needed, based on symptoms or concerning physical examination findings. Across series, rib fractures seem to occur most often within the first 2 years after radiation. Information is limited regarding the symptomatic or asymptomatic nature of the fractures.

A multitude of factors could explain the heterogeneity in the rates of rib fracture. Regarding treatment technique, 2 beams were used for PBS treatment in our series, rather than a single-beam approach, as reported by Jimenez et al [24]. In addition, our patient population was younger, with a difference in the median age of 10 years. Our series includes a larger percentage of Black women (26%), who generally have higher bone mineral density than White women. However, neither race nor bone density measured by DEXA scan was associated with rib fracture on univariate analysis [26].

In breast cancer radiation therapy (photon or proton), ribs are not routinely contoured as an organ at risk. Some contouring atlases recommend the inclusion of the ribs in the CTV volume for postmastectomy radiation and locally advanced primary disease treated with breast-conserving surgery [27]. It may be informative to contour ribs prospectively to assist in data acquisition regarding rib dose and subsequent toxicity. Plans could be adjusted to limit hot spots on the ribs or establish a rib dose constraint. However, the clinical relevance may be limited, given the low rate of symptomatic rib fractures.

A significant limitation in assessing the incidence of rib fractures after radiation is the variability of postradiation surveillance imaging. National Comprehensive Cancer Network guidelines do not endorse routine surveillance imaging for asymptomatic patients. Therefore, the timing and extent of postradiation imaging are highly variable, based on patient symptoms, examination findings, and disease characteristics. All rib fractures in this series were identified on surveillance imagining; the only symptomatic patient had negative imaging at the time of symptom onset but positive imaging subsequently. Therefore, additional asymptomatic rib fractures may have occurred in patients who did not undergo surveillance imaging, resulting in the potential underestimation of rib fractures and complicating interseries comparisons. This limitation is present across most studies that report postradiation rib fractures. It is notable, however, that in this series, the rate of symptomatic rib fractures is very low at < 1%, and rib fractures occur out of the radiation field at a nonzero rate. The variability in rib fracture rates amongst proton series may be attributable to the relatively small patient numbers. Results of the RADCOMP trial [28] may provide the best data for this endpoint, given the large cohort, long follow-up, and randomized trial design, which will allow a direct comparison of the incidence of rib fractures by modality. Other limitations of our study include short follow-up and nonrandomized design.

Although a proton beam has a higher biological dose deposited at the end-of-range than the assumed constant RBE = 1.1, the 3-year rates for symptomatic and any in-field rib fractures in our series remain low at 0.4% and 3.7%. As in the photon series, the asymptomatic rib fracture rate may be underestimated owing to the lack of routine surveillance imaging. However, patients experiencing symptomatic rib fractures after proton therapy for breast cancer are rare.
